# Supplementary material for: Are carbon emissions trading and green financial instruments synergistic? -Comprehensive quantitative research based on content analysis
Source: PLoS One. 2024 Mar 7;19(3):e0298601. doi: 10.1371/journal.pone.0298601 (PMC10919601; doi:10.1371/journal.pone.0298601)
Supplement: S1 Data — (ZIP) [file pone.0298601.s001.zip › Data/地级市Z_F工作报告.zip!/║ú─╧/╚2╤╟╩╨/└┤╫╘╣1⁄2╓┌║┼í╛┬φ┐╦╩2╛▌═°í┐_2016.docx]

各位代表：

现在，我代表市政府向大会报告工作，请予审议，并请市政协委员和其他列席人员提出意见。

2015年和“十二五”工作的回顾

2015年，市政府在省委、省政府和市委的正确领导下，在市人大、市政协的监督支持下，坚持稳中求进、改革创新，努力推动经济社会的发展和城市品质的提升，取得了新的成绩。全市实现生产总值435亿元（含农垦），同比增长8.1%；完成固定资产投资706亿元，增长12.1%；完成社会消费品零售总额181.7亿元，增长8%；地方一般公共预算收入达到88.9亿元，增长11.1%；城镇、农村常住居民人均可支配收入分别为28782元和12228元，分别增长7.2%、8.4%。一年来，我们主要加强了以下工作：

一、统筹推进“双修”“双城”试点和“城市治理管理年”活动

去年6月，三亚被国家住建部列为全国292个地级市中唯一的“双修”“双城”综合试点城市。我们以此为契机，结合“城市治理管理年”活动，积极加快试点步伐，坚持让绿于民、让路于民、让景于民、让海于民、让田于民，打好交通环境、旅游市场、涉海旅游、市容市貌、违建拆除五大关键战役，提升了城市形象和品位。开展了“双修”“双城”6项规划编制和11个近期施工类项目的规划设计，分别置换两处房地产项目用地，改建为市民果园和红树林公园，凤凰路、榆亚路、迎宾路景观提升工程等首批施工类项目进展顺利，我市首段地下综合管廊开工建设。完成了三亚湾人工补沙项目，全面实施三亚湾原生植被保护及生态恢复工程。强力推进海岸带保护与开发核查，清理整改问题114项。三亚获评全国第二批国家级海洋生态文明建设示范区。抱坡岭等山体恢复效果明显。全市完成补植养护“退果还林”3509亩。去年新建污水管道58公里，疏通清理市区污水主管道220公里，整治沿河排污口76处，清理整治畜禽养殖、屠宰场等沿河重点污染源131家，安装移动式污水处理站6处，新建、扩建污水处理厂、污水提升泵站的工作正在推进，三亚河保护区红树林修复已经启动，城市内河治污取得明显进展。人民日报两次在头版报道三亚的生态治理成效。去年全市共拆除违建388.6万平方米，同时积极开展“两普查、两规划、一报建”工作，强化了控违基础。完成了年初确定的“严查存量，严禁增量”的打违控违目标任务。声势浩大的打违行动得到了包括央视在内的主流媒体和广大人民群众的普遍称赞。积极创建全国旅游标准化试点城市，加强旅游市场整治和监管，启动运行市民游客中心，并线运行12301旅游服务热线和12345政府服务热线，成立国内首支旅游警察队伍，荣获“2015中国十大社会治理创新”奖，全国旅游市场秩序整治工作现场交流会在三亚召开。积极开展广告牌匾整治，规范沿街店铺招牌，推动拆墙透绿、拆违建绿，整治占道经营，狠抓环境卫生，市容市貌得到改善。新建成一批市政道路和桥梁工程，红沙隧道实现通车，“海上巴士”投入试运营，西线旅游铁路动工，改造、新建了一批停车场，新开通和优化调整35条公交线路，新投放了500辆出租车，并引进了滴滴打车、神州专车等服务项目。过去的一年，“双修”“双城”试点工作的扎实推进和“城市治理管理年”活动的有效开展，使三亚的市容市貌和旅游服务市场秩序发生了巨大变化，得到了社会各界的普遍赞扬和省委、省政府的充分肯定。

二、加快推进结构调整

通过供地结构调整，促进产业结构调整，去年全市供应国有建设用地3119亩，商品住宅项目用地仅占3.6%。旅游产业规模和效益稳步提升，全年接待过夜游客1495.7万人次，旅游总收入302.3亿元，分别增长10.6%和12.1%；凤凰机场旅客吞吐量1619万人次，增长8.4%；去年新开通国际航空航线11条，入境游客降幅逐月收窄，中高端游客比例上升；三亚国际免税城接待顾客482.6万人次，营业收入42.3亿元，低空旅游接待游客增长140.8%，会展旅游、婚庆旅游、文化旅游等业态保持较快增长。全市商品房销售面积100.7万平方米，增长7.1%。农业农村经济平稳发展，热带高效农业规模壮大，划定南繁基本农田保护区9.98万亩，全市实现农业总产值92.3亿元，增长5.3%。兴业银行三亚分行即将开业，用友新道科技股份有限公司实现“新三板”上市，金融业增加值50.2亿元，增长20.7%，在GDP中所占比重达到11.6%。三亚的产业结构调整取得可喜进步，房地产业在第三产业中的比重正在逐步降低。

三、全力抓好项目建设

坚持从保障用地供应、提高审批服务效率、突出区级属地管理、拓宽投融资渠道等方面入手，多措并举推进项目建设。特别是去年9月下旬以来，扎实开展“投资项目百日大会战”，推动136个项目开工建设，完成投资40.9亿元，有力保障了投资增长。去年市重点项目完成投资461.3亿元，完成年度计划的108.9%。凤凰机场T2航站楼、西环高铁三亚各站点投入运营，凤凰岛国际邮轮港一个15万吨级的泊位投入试运行，海棠湾亚特兰蒂斯等项目加快建设，中兴通讯三亚研发中心、用友新道科技股份有限公司总部、三亚国际旅游管理学院等项目实现新开工，海棠湾梦幻不夜城、世界手工艺理事会国际交流中心、恒大国际合作医院、人寿健康中心、中电信息安全中心等优质产业项目实现落户，新机场项目启动前期工作。以邮轮港为载体引进了中交建、港中旅等企业，中交建新成立的海南投资公司和邮轮中心在三亚注册落地。

四、扎实做好民生工作

全年民生支出89.9亿元，增长18.4%，占一般公共预算支出的76%。全年城镇新增就业人员3.2万人，农村劳动力转移就业1.4万人，城镇登记失业率为1.63%。完成了机关事业单位养老保险制度改革。全市城镇从业人员五项保险和城乡居民基本医疗保险登记参保人数稳步增长。实施城乡居民大病保险制度，扩大基本医疗保险特殊病种范围，并相应提高了各病种待遇标准。制定棚户区改造三年计划，并争取到国开行贷款支持，2015年已落实一期18个项目的贷款资金，完成棚改9842户。海棠区将近2000户村民搬进了位于林旺南、林旺北、龙江、龙海风情小镇的新房，产城融合的新型城镇化建设在海棠湾得到了扎实有效的实践。海南热带海洋学院挂牌成立，136所公办义务教育学校完成标准化建设并通过省级评估验收，三亚中等职业技术学校升格为三亚技师学院。中医院三期工程竣工，市人民医院急诊综合楼开建，哈尔滨医科大学鸿森医院、国康医院等一批民营医院正在加快建设。美丽乡村试点扎实推进，农村饮水安全、扶贫攻坚、农田水利、病险水库加固、农村公路通畅工程与桥梁改建维修等工作取得新成效。认真抓好安全生产工作，及时排查调处矛盾纠纷，妥善处理信访问题，深入开展打击“两抢一盗”、“打黑除恶”等专项行动，社会保持和谐稳定。

五、继续深化改革开放

“多规合一”工作扎实推进，通过“多规合一”明确了三亚未来重点发展的9个产业、14个产业园区、8个产业小镇，划定了相应的生态红线，并对经济社会发展规划、城乡规划、土地利用规划以及林地、农地等规划存在的矛盾进行了清理。务实规划“大三亚”旅游经济圈，强化与陵水、乐东、保亭的协同合作。继续推进行政审批制度改革，初步精简了396项前置审批条件和申请材料，实现了政务服务事项清单式管理；通过完善并联审批、实施审批代办等措施，提升了项目审批效率。工商注册制度便利化改革稳步推进。率先组建不动产登记机构，颁发全省第一批不动产产权证书。新设各区正式挂牌运行，并分两批从市级下划人员，推动权力下放、责任下划、人员下移。在省委、省政府的关心和推动下，离岛免税购物政策再次实现放宽调整。成功举办了2014-2015沃尔沃环球帆船赛三亚站的比赛、2015世界拳击联赛等文体赛事活动，世界小姐总决赛重返三亚，三亚的知名度和美誉度进一步提升。

六、着力加强政府自身建设

认真开展“三严三实”专题教育，转变工作作风，提升工作效能，加强廉政建设，查处了一批违法违纪案件。全面推行部门预决算和“三公”经费预决算的公开，深化了政务公开工作。切实加强审计监督，加大对预算执行和政府投资的审计力度，保障了公共财政资金的安全运行。认真执行市人大及其常委会的决议决定，自觉接受市人大及其常委会的法律监督、工作监督和市政协的民主监督，全年办复市人大代表建议116件、政协提案159件，办复率100%。

积极开展国防教育、国防动员、民兵预备役工作，扎实推进双拥共建工作，军政军民团结进一步巩固。民族宗教、外事侨务、人口计生、档案、史志、海防、口岸、打击走私、防震减灾、食品药品安全、气象、人防等工作取得新成绩。刚刚过去的春节黄金周大考，在各级各部门和全市人民的共同努力下，三亚再次向省委、省政府和来自全国各地的游客交出了一份满意的答卷。

各位代表！过去的五年，我们积极应对复杂形势，努力克服各种困难，三亚发展实现了新的跨越：一是经济实力快速增强。全市生产总值从2010年的231亿元增加到2015年的435亿元，接近翻一番，常住人口人均生产总值从36372元增加到58361元；地方一般公共预算收入从42.2亿元增加到88.9亿元，增长了一倍以上；社会消费品零售总额从63.7亿元增加到181.7亿元，增长了将近两倍；城镇常住居民人均可支配收入从17758元增加到28782元；农村常住居民人均可支配收入从6502元增加到12228元；五年累计完成固定资产投资从“十一五”期间的849.2亿元增加到“十二五”期间的2647.5亿元。二是旅游服务接待能力显著提升。全市新增高端度假酒店17家，三亚国际免税城、三亚千古情等一批新的特色旅游景区建成运营。海棠湾“国家海岸”开发建设取得显著成绩，一个世界级的综合旅游度假区正在形成。三是基础设施加快建设。绕城高速公路、西环高铁、南山滚装码头、崖州中心渔港等一批重要基础设施项目建成投用，机场、邮轮港等设施加快扩建，城乡电网、供水管网、天然气管网和光纤网络进一步完善。四是公共服务体系不断完善。“十二五”期间，财政民生投入从“十一五”时期的108.2亿元增加到337.5亿元。全市各级各类学校比2010年增加44所，学前三年毛入园率和高中阶段毛入学率分别提高6.4和10个百分点。解放军总医院海南分院建成开诊，有两家市属医院被新评为三级甲等医院，行政村卫生室和社区卫生服务网络覆盖率达到100%。社会保障在较短时间内基本实现全覆盖。五年来共建成保障性住房10202套，完成农村危房改造8508户，18334人脱贫。五是社会治理管理体系更加健全。经过不懈的努力，有效遏制了违建蔓延的势头，环境卫生、旅游服务市场、交通等方面的一些顽疾问题得到了解决，城市管理整体水平显著提升。社会治安明显好转，“两抢一盗”等刑事案件的发案率大幅下降。全社会矛盾调处能力明显提升。六是生态环境得到优化。生活垃圾焚烧发电厂一期工程、渗滤液处理站改扩建工程以及餐厨废弃物处理厂等项目建成运行，垃圾收集转运体系更加健全。新建城镇污水处理厂两座，全市污水处理能力从每天18.5万吨提升到每天22.5万吨，新建雨水、污水管道267公里。加强水污染治理和生态修复，因城市规模扩大、人口增加而造成的生态环境破坏的势头得到了有效遏制。完成了“十二五”节能减排目标。七是改革开放深入推进。“撤镇设区”工作顺利完成。大力精简行政审批事项，截至去年底我市市直单位和省垂管单位保留的行政审批事项已减至181大项。航油保税、邮轮边境旅游异地办证、外籍邮轮特许开展多点挂靠等政策获得批准。三亚被列为国家“一带一路”海上合作战略支点城市。

各位代表！能够取得这些成绩，离不开省委、省政府和市委的正确领导，离不开市人大、市政协和社会各界的大力支持，更是全市人民团结拼搏的结果。在此，我谨代表市政府，向全市人民致以崇高的敬意！向人大代表、政协委员、各民主党派、人民团体和社会各界人士，向中央和省驻三亚各单位，向驻市部队、武警官兵、公安干警，向所有关心支持三亚发展的国内外朋友，表示衷心的感谢！

在肯定成绩的同时，我们也清醒地认识到，三亚在发展中还存在不少矛盾和问题。经济总量小、产业结构单一、抗风险能力弱的局面没有根本改变，结构调整的任务还很繁重；生态环保压力增大，城市基础设施建设和治理管理仍需加强；一些部门职能和作风转变不到位，行政效能仍需提升，等等。对此，我们将高度重视，认真解决。

“十三五”的奋斗目标和主要任务

按照中央、省委省政府和市委的部署，到“十三五”期末，三亚将全面建成小康社会、初步建成国际化热带滨海旅游精品城市。要实现这两个目标，我们必须坚持“四个全面”战略布局，牢固树立创新、协调、绿色、开放、共享的发展理念，坚定不移走科学发展、绿色崛起之路，并努力完成以下五项主要任务：

一、产业结构极大优化，三亚成为旅游产品最优异的城市

按照第一产业“增效调优”、第二产业“集中做强”、第三产业“转型跨越”的总体要求，积极推进供给侧结构性改革，并突出抓好旅游业这个龙头，打好热带高效农业这张王牌，加快形成“旅游+”产业发展新格局。到2020年，全市生产总值超过600亿元，年均增长7%（按可比价计算），人均生产总值达到77600元；地方一般公共预算收入超过130亿元；固定资产投资突破1100亿元；城镇常住居民人均可支配收入突破40000元，农村常住居民人均可支配收入达到18000元；以旅游业为龙头，涵盖医疗健康、会展、金融、总部经济、文化体育、教育科技、创新创意等业态的现代服务业得到快速发展，抗风险能力强、可持续发展能力强的新型产业体系基本建成，旅游服务业的综合实力显著增强，水平全面提升，业态更加丰富。

二、城市功能极大完善，三亚成为风貌形态最优美的城市

到2020年，三亚新机场初步建成，国际邮轮母港全面建成，海陆空无缝衔接、方便快捷的公共交通网络体系全面建成。“双修”“双城”试点工作成效显著，棚户区改造任务基本完成，城市道路体系、绿化体系、夜景灯光体系基本完善，各类市政和服务设施的精品化、低碳化、智慧化、国际化改造基本到位，河流水系连通、湿地再造以及主要的山体修复任务基本完成，独具特色的城市风貌更加彰显，美丽三亚更加迷人。

三、环境质量极大提升，三亚成为生态环境最优越的城市

始终坚持生态立市的基本原则，到2020年，生态城市建设取得重要进展，三亚的城市生活垃圾无害化处理率、餐厨垃圾集中处理率均达到100%，城镇污水集中处理率达到90%以上，城市内河水质、海水水质全部达标，省政府下达的六项整治任务全面完成，生态环保立法工作成效显著，单位GDP能耗继续下降，主要污染物排放总量严格控制在省下达的计划目标内，全市森林覆盖率保持在68%，空气质量优良天数比例保持在95%以上，三亚的天更高、海更蓝、水更清、山更绿。

四、治理管理极大加强，三亚成为综合秩序最优良的城市

秉持“治理管理与发展同等重要”的理念，持续推动城市治理管理，到2020年，三亚基于法制化、信息化的环境卫生、交通、生态、旅游服务市场、治安等方面的治理管理体系基本完善，城乡规划建设管理体系更加规范成熟；政府对市场和社会的管理与服务能力进一步提高，市场在资源配置方面的决定性作用进一步彰显；市场充满活力，人民安居乐业，社会和谐稳定。

五、公共服务极大改善，三亚成为服务资源最优裕的城市

大力引进国内外优质的教育资源、医疗资源、文化资源，并通过“互联网+”战略，积极开展教育扶贫、医疗扶贫、文化扶贫，让优质资源辐射到每一个社区、每一个村庄，惠及每一个家庭、每一个市民，城乡居民的综合素质得到全面提高。高标准建设博物馆、美术馆、音乐馆、大剧院、体育中心、青少年活动中心等公共文化体育设施，常态化、机制化地举办各类音乐节、艺术节以及文体赛事。到2020年，三亚成为优质教育资源、医疗资源、文化资源人均占有率最高的城市之一。三亚既是外来游客的度假天堂，又是本地人民的幸福家园。

为完成上述任务，我们要实施十大工程、十项计划，确保各项工作落到实处，各项任务如期完成。十大工程包括生态修复工程、城市修补工程、棚户区改造工程、新机场工程、邮轮港工程、轨道交通工程、海绵城市建设工程、地下综合管廊工程、公共文化、体育及政务服务设施工程、美丽乡村建设工程；十项计划包括“菜篮子”计划、产业发展计划、全域旅游发展计划、国际化进程推进计划、智慧城市和低碳城市发展计划、教育发展计划、医疗卫生发展计划、精准脱贫和城乡居民增收计划、社会治理管理体系建设计划、人才队伍建设计划。

2016年的工作安排

2016年是实施“十三五”规划的第一年，做好今年的各项工作，对于“十三五”开好局、起好步具有重要意义。根据省委、省政府的要求和市委的部署，今年市政府工作的总体思路是：全面贯彻党中央、国务院和省委、省政府的精神和要求，按照市委六届六次全会的部署，紧紧围绕“四个全面”战略布局，牢固树立五大发展理念，扎实推进供给侧结构性改革，积极融入“大三亚”旅游经济圈，以“多规合一”为基础，以十大工程、十项计划为抓手，做实做强三亚经济，做大做精三亚旅游，做优做美三亚城市，进一步加快全面建成小康社会和国际化热带滨海旅游精品城市的步伐。今年的主要预期目标是：生产总值增长7%-7.5%；固定资产投资增长10%；社会消费品零售总额增长8.5%；地方一般公共预算收入增长8%；旅游总收入增长11%；城镇常住居民人均可支配收入增长7%，农村常住居民人均可支配收入增长8.5%；居民消费价格涨幅控制在3.5%以下；完成省下达的节能减排降碳控制目标。

一、推动十大工程，夯实发展基础

（一）生态修复工程。继续实施三亚湾原生植被种植工作，推进海岸线保护，实施亚龙湾青梅港红树林自然保护区建设。扎实推进城镇内河（湖）水污染治理三年行动，科学开展红树林生态保护，力争年内实现三亚河水质达标。加强山体修复和森林资源保护，加快25度以上坡地的“退果还林”进度，推广生态果园，确保年内在全市全面推开。完善体制机制，加强大气污染监测与防治，严控城市扬尘污染，年内要做到应控尽控。结合三亚的实际，通过实施土壤地力提升和化肥、农药减量等措施，认真开展土壤环境综合治理专项行动，从严控制农业面源污染。

（二）城市修补工程。积极推进城市污水处理系统建设，加快启动红沙污水处理二厂建设、新城水质净化厂二期扩建，继续推进移动式污水处理站建设，年内要建成20个，同时要新建污水管道67.9公里，新建污水提升泵站6座，并完成两座原有泵站的扩容。继续完善垃圾回收处理系统，加快建设年处理能力为50万吨的建筑废弃物综合利用厂并在年内投产；争取年内完成垃圾焚烧发电厂二期工程，日处理能力达到1200吨；采取切实有效的措施收集餐厨垃圾，保证日处理能力100吨的餐厨废弃物处理厂常态化运行。积极推进市政道路建设，加快实施胜利路改造以及新建龙岭路、海螺路等道路桥梁工程，完成跃进桥、月明桥建设和225国道改扩建，高标准推进绕城高速公路服务区规划设计。继续在全市选点建设停车楼，加强对各类商场和小区停车设施配置的刚性要求，加快完善全市的停车系统。加快实施各项园林绿化工程，继续推进“一湾两河三路”的绿化景观提升，年内完成城市绿线控制规划编制工作，强化绿地管护。以三亚湾城市立面、三亚河两侧及城市主要制高点全景式灯光为主要载体，高标准、高起点、规范化建设全市夜景灯光系统，年内基本完成。以规范全市的店招店牌和广告牌为主要抓手，以解放路的立面改造为补充，规范城市的街道立面。近期，市政府将出台规范城市建筑风貌、城市主色调、城市天际线的相关规定，积极筹备好国家住建部在三亚召开的城市“双修”现场会。

（三）棚户区改造工程。今年要争取完成吉阳区的东岸村、榕根村，天涯区的海坡村，海棠区的江林村、龙海村的拆迁工作，并完成项目的招商落地；启动吉阳区的月川村、临春村，天涯区的西瓜村、芒果村，海棠区的藤海村等棚户区改造。要加快启动崖州区南山及中心渔港安置区附近的棚户区改造和土地整理工作。要把棚户区改造作为完善城市功能、优化城市形态、改善人居环境、提升城市品质的重要民生项目来抓。

（四）新机场工程。积极协助省政府完成国家相关部委批复同意三亚新机场建设的手续，并加快推动采石场相关手续的办理，争取早日全面开工，年内完成40亿元以上的投资。同时，要加快凤凰机场三期扩建，规划布局和建设全市的直升机线路和停落场点。

（五）邮轮港工程。年内完成四个码头的建设，启动航站楼及相关配套设施建设，完成邮轮港配套的交通体系、商业服务设施的规划设计。亚洲最大邮轮母港的雏形将呈现在世人面前。完成“海上巴士”所需的南山、天涯海角、海棠湾等码头建设，并全线开通运营。

（六）轨道交通工程。今年要积极争取中国铁路总公司的支持，启动海棠湾动车站的建设，全面完成西线旅游铁路建设并投入运营。全面启动有轨电车示范段建设，并启动市区主线的前期工作。年内启动动车站交通枢纽综合体建设。

（七）海绵城市建设工程。今年将完成白鹭公园的水系连通，基本完成金鸡岭桥湿地公园、丰兴隆桥湿地公园的建设以及海棠湾的内河水系整治。全面启动东岸湿地建设以及三亚东西河河道及两侧的景观整治，启动三亚河西部水系连通工程，适时启动宁远河和藤桥河的水系整治工作。

（八）地下综合管廊工程。在完成海棠湾示范段建设的同时，加快推进胜利路等新线路的建设。成立地下综合管廊运营公司，出台运营管理办法，规范管廊运营管理。进一步探索PPP模式，引入社会资本，加快推进全市的综合管廊建设。

（九）公共文化、体育及政务服务设施工程。抓紧策划建设市档案馆、博物馆、美术馆、规划馆、青少年活动中心以及市政务服务中心等项目。今年要完成规划选点，做好用地安排，并全面启动规划设计工作。要进一步扩大广播电视在偏远地区的覆盖面。

（十）美丽乡村建设工程。对中短期内城镇化不能覆盖到的乡村，按照“望得见山，看得见水，记得住乡愁”的理念和基础设施配套、文化特色突出，以及发展全域旅游的要求，全面整合政府资源，积极调动社会资源，统一规划设计，统一实施建设。今年全面启动规划设计，并选出试点村庄进行改造。

二、实施十项计划，全面转型升级

（一）认真实施“菜篮子”计划。落实好省政府“菜篮子”工作十项措施。探索建立国有蔬菜公司。加快推进公益性蔬菜销售点全覆盖，扶持守信用、有实力的蔬菜企业进入公益性蔬菜销售点进行直销，减少流通环节，压低批零差价，保证菜品质量。同时，要切实落实常年蔬菜生产基地，实现产销对接，建设政府可掌控、能调剂、有保障的蔬菜储存、调运、供应体系，确保让广大市民和游客常年吃上平价菜、放心菜。

（二）加快实施产业发展计划。坚持“以亩产效益论英雄”，围绕9个重点产业，发挥14个产业园区和8个产业小镇的载体作用，大力引进优质企业和产业项目，优化产业结构。积极发展健康产业，加快建设海棠湾已经落实的恒大国际合作医院等5个健康产业项目，尽快完成半岭温泉健康产业园规划并积极对外招商。市政府成立健康产业发展引导基金，在博鳌乐城国际医疗旅游先行区注册成立公司，以充分利用国家的优惠政策，助力国内外更多有实力的健康养生机构落户三亚，形成健康产业集群，争取用较短的时间把三亚打造成为健康养生的胜地。以创意产业园为主要支撑，进一步完善园区的教育、医疗等配套设施，紧紧抓住北京疏解非首都功能和深圳产业溢出效应的机遇，加大招商力度，承接产业转移，使三亚的创意产业尽快上规模、上水平。以三亚河口附近及凤凰岛周边区域为重点，加快发展邮轮、游艇产业，争取年内完成水居巷拆迁，完成南边海及老码头区域的开发建设规划。在省委、省政府的指导支持下，争取国家在邮轮游艇产业发展方面给予三亚更多的优惠政策，把三亚打造成为国内实力最强的邮轮游艇产业基地。年内启动建设海棠湾、三亚湾的会展中心，成立专职会展管理机构，引进有实力的会展公司，推进会展产业发展，使三亚成为最活跃的会展城市之一。做好海南国际旅游贸易博览会的承办工作。与中科院深海所、遥感所积极对接，加快推动遥感和海洋科技产业园的开发建设，推动三亚科技产业加快发展，争取上半年完成规划，下半年启动建设。以市南繁院为基础，积极推动海南三亚国家农业科技园区建设。继续引进各类金融机构，支持金融产品和服务创新，促进金融产业与经济发展协调共进。积极推进分时办公，发展总部经济，加快推动物流产业园、地方小工业园的规划建设。认真落实省政府关于加强房地产市场调控的要求，加大去库存力度。加快规划完善迎宾路等片区的商业、医疗等设施，充分发挥房地产交易市场和房地产促销信息平台的服务功能，提高办税、办证等服务水平，规范中介机构和二手房市场管理，促进房地产业平稳健康发展。

（三）分区推进全域旅游发展计划。继续严把项目入口关，高标准建设海棠湾综合旅游度假区，今年君悦、艾迪逊、皇冠、红树林等四家酒店投入营业，梦幻不夜城、水稻国家公园等一批重点项目开工建设，加快亚特兰蒂斯、四季酒店、W酒店等项目的建设进度，并积极引进更多的文化、旅游项目落户海棠湾，使海棠湾的综合旅游接待能力进一步增强。加快推进亚龙湾休闲外交基地建设，按照精品化、高端化的要求，对亚龙湾的业态进行调整。以大东海、鹿回头广场改造为契机，推动大东海景区的升级改造。以有轨电车建设为契机，加快推进三亚湾的业态调整和整体提升，打造中心城区的精品商业旅游区。进一步规范大东海、三亚湾的海上旅游项目，加快推动夜游三亚湾旅游文化项目的提档升级，使海上旅游成为三亚更亮丽的品牌。加快谋划天涯海角景区的改造和扩展，丰富景区的内涵。以南海佛学院建设为契机，高标准规划南山佛教文化景区，把南山打造成为世界一流的佛教文化旅游胜地。尽快完成中心渔港的项目回收，整体规划建设渔港小镇。坚持“交通互联、产业互补、资源共享、发展同步”，实质性推进“大三亚”旅游经济圈建设。

（四）加快实施国际化进程推进计划。通过引进国际服务机构，对标国际服务标准，举办国际文化体育活动，开辟国际空中海上航线，打造国际门户枢纽机场和国际邮轮母港，加快推进三亚的国际化进程。2016年要着力引进健康养生机构，高标准推进海上歌剧院等项目规划设计，积极引进国际品牌的文化体育活动和论坛会展，开辟更多的欧洲、亚洲空中航线和邮轮航线，努力让沃尔沃环球帆船赛、国际拳击赛、世界小姐总决赛、网球赛、马拉松赛等赛事成为常驻三亚的赛事，推动三亚财经国际论坛、三亚中国企业国际化论坛、能源论坛、健康论坛成为影响力更大的国际论坛，积极策划国际电影节、邮轮节、音乐节、艺术节等国际化节庆活动。按照国际化的标准对三亚的主要道路和两侧景观、旅游厕所、海鲜排档、水果店、餐馆、农贸市场、特产店、旅游纪念品店以及各类商业铺面进行全面的规范提升，培育懂外语、素质高的服务业队伍。

（五）加快实施智慧城市和低碳城市发展计划。全面提高信息基础设施建设水平，年内实现旅游景区和主要公共场所WIFI全覆盖。从政务、交通、城管、旅游、医疗等方面的信息共享平台和大数据应用入手，坚持“互联网+”战略，促进互联网与其他产业融合发展，积极打造智慧城市。狠抓节能减排，强化重点行业和重点单位监管。继续推广节能技术和新能源产品，加大力度在公务用车、公交车、出租车等领域推广新能源汽车。积极发展低碳绿色建筑，将低碳绿色建筑的指标作为规划建设的刚性指标来落实。今年要完成智慧城市和低碳城市的发展规划，并积极向前推进。

（六）积极推进教育发展计划。进一步提升全市义务教育学校标准化建设水平，推动义务教育均衡发展。扎实推进教育信息化建设，扩大优质教育资源覆盖面。今年要研究确定刚性指标，加大优秀校长、教师的培养和引进力度，争取在较短的时间内使三亚基础教育的师资队伍水平整体显著提升。积极开展与先进地区和名校的基础教育合作，按照一区一所有实力的中小学进行布局，积极与清华大学、人大附中、华南师大附中、西南大学等学校对接洽谈，争取年内落地。加快公立幼儿园建设，扶持优质民办幼儿园发展，争取在较短的时间内使三亚的学前教育水平达到国家的要求。积极推动省市共建三亚学院，推进海南热带海洋学院建设，加快推动三亚国际旅游管理学院建设、三亚海航学院迁建，使三亚的高等教育发展水平再上一个新台阶。继续大力发展职业教育，促进就业创业。

（七）加快实施医疗卫生发展计划。积极引进国内外一流的医疗机构，按照一区一所名医院进行布局，近期抓紧与华西医院进行对接。通过建立名医工作站等措施，更好地发挥候鸟名医的作用。继续推进市人民医院急诊综合楼建设和市疾病预防控制中心搬迁等工作，进一步完善医疗卫生基础设施。以三甲医院为依托，规划提升医疗急救能力。通过人才培养、送医下乡等措施，强化基层医疗力量，加强“互联网+”在医疗服务中的应用，尽快地使优质医疗资源向基层延伸。实施一对夫妇可生育两个孩子政策，提高优孕优生优育服务水平。

（八）扎实推进精准脱贫和城乡居民增收计划。积极落实国家和省的扶贫政策，引入社会资源，落实领导责任，精准扶贫方式，加大力度推进，确保三亚的贫困户年内全部脱贫。认真抓好农田水利设施建设，继续做好农村饮水安全工作。推动国家南繁基地、冬季瓜菜基地建设。继续培育农业龙头企业和农民专业合作社，推广先进农业技术和农业标准化种植，抓好农产品质量安全工作，打造特色农产品品牌，加快农村电商发展。积极促进农旅融合，结合特色产业小镇和水稻国家公园等项目建设，继续发展休闲农业与乡村旅游，探索民宿建设标准与发展模式。继续引导和扶持大吨位渔船更新改造，组织赴三沙海域开展常态化生产，继续推动以海洋牧场为主的休闲渔业发展，推进渔业转型升级和渔民增收。落实就业、创业扶持政策，鼓励小微企业发展，大力开展劳动力职业技能培训，加强就业、创业公共服务。

（九）稳步推进社会治理管理体系建设计划。认真开展整治违法建筑三年攻坚专项行动，保持打违高压态势，重点拆除连片违建，巩固深化拆违成果，争取今年拆除300万平方米以上的违建；继续深入开展“两普查、两规划、一报建”工作，加快推进农村地区规划建设的规范管理。持续打击旅游市场“五黑”、非法“一日游”、价格欺诈、商业贿赂等行为，加强涉海旅游监管；发挥好市民游客中心、旅游警察、旅游巡回法庭和12345、12301热线的作用，进一步提升涉旅事件处理效率，完善常态化管理机制。加强交通管理、疏导，对交通违章行为实行严管重罚，特别是要想方设法规范电动车通行秩序，力争年内实现全市的停车规范管理。进一步强化竞争机制和监管机制，提高城乡环境卫生常态化管理的质量；建立健全社区、公安、城管、环卫“四位一体”的网格化管理机制，切实落实“门前三包”责任制。上半年完成数字城管系统和城市管理服务热线的整合。进一步推进“强区扩权”，加强社区自治能力建设，加快完善区直管居（村）扁平化管理模式。深入开展法制宣传教育活动，建立健全社会诚信体系；不断强化安全生产责任制，加强重点行业领域安全隐患排查和专项整治，严防重大事故；完善社会治安防控体系，加强社会治安综合治理，创建“和谐三亚”。

（十）坚持实施人才队伍建设计划。努力创造人才优势，形成发展优势，制定和落实好年度人才引进与培养计划。创新人才户籍迁移、家属就业、子女入学等政策，充分利用三亚市国际旅游岛人才创业基地和产业园区的平台作用，积极引进各领域的高层次人才和实用型人才，大力培养和留住本地人才。通过建立候鸟人才库等措施，创造性地用好教育医疗、文化艺术等各个领域的候鸟型人才。

三、践行“三严三实”，建设人民满意的政府

（一）人民满意的政府首先必须是廉洁公平的政府。我们一定要坚决反对腐败，坚决反对“吃拿卡要”，坚决反对懒政怠政，坚决反对不干事、不担事。政府的各部门和各类工作人员都要自觉接受广大人民群众的监督，政府将通过微信等信息平台随时听取社会各界对政府各部门和各类工作人员的意见和评价，以不断地改进我们的作风。我们要高度重视和自觉运用审计监督，及时发现和解决问题，不断提升政府自身的纠错能力。要自觉接受市委及其纪检部门的监督，坚决查处各类违法违纪案件，坚决纠正损害群众利益的不正之风。

（二）人民满意的政府必须是真抓实干的政府。实施十大工程、十项计划，是推进三亚发展转型升级、全面建成小康社会和国际化热带滨海旅游精品城市的有效途径和抓手。我们必须按照“三严三实”的要求，抓好每一个工程、每一项计划的落实，要明确每一项工作的时间进度和责任单位。在实施的过程中，要时刻注意回应社会各界的关注，让广大人民群众放心和满意。要强化督查督办，对那些在推动征地拆迁、打击违建、棚户区改造等各项工作中不作为、乱作为的人，坚决进行问责。

（三）人民满意的政府必须是依法行政的政府。我们要依法推动征地拆迁、项目建设，依法管理城市、监管市场，依法严厉打击各类违法犯罪活动，对违法建设、破坏生态环境、欺客宰客、“两抢一盗”、违法行驶等行为实行“零容忍”。要认真执行市人大及其常委会的决议、决定，自觉接受人大、政协监督，提高人大代表建议和政协提案的办理质量，积极配合市人大开展各项立法工作。

（四）人民满意的政府必须是改革创新的政府。我们要加快完成全市“多规合一”的各项收尾工作，积极争取省相关部门的支持，抓紧研究细化“多规合一”技术标准、实施管理办法，加快相关信息平台、数据库和系统建设，实现“一张表”审批，使“多规合一”的成果尽早在实际工作中得到广泛应用。要继续深入推进行政审批制度改革，在省有关部门的指导下，完成市、区两级单位“责任清单”和“权力清单”编制，继续清理、下放审批服务事项，规范中介服务行为；推行审批综合受理、公共服务集中办理以及流程标准化，进一步探索承诺制办理；在海棠湾现代服务业产业园开展区域评估评审试点。要积极参与新一轮农垦改革，抓紧做好南新农场整体移交三亚管理的承接工作，同时采取多种方式，主动承接其他国有农场需要移交的社会管理和公共服务职能；积极支持南田国家现代农业示范区、三亚凤凰谷乡村文化旅游区、南滨国家南繁种业园区等农垦产业园区的建设发展；结合“多规合一”，促进各农场在规划布局、土地利用、产业发展、治理管理等方面与三亚经济社会发展的总体布局相协调。积极探索通过设立产业发展基金、引入社会资本推动市属国企重组以及通过PPP方式引入社会资本参与城市建设等途径，促进三亚的基础设施建设以及旅游、医疗健康等优势产业的快速发展，使三亚的资源优势尽快转化为发展优势。与此同时，我们要密切监控政府的债务风险，将政府债务控制在安全、合理的区间。继续实施公立医院改革，完成国家公立医院改革试点各项任务。积极推进“多证合一”改革，完成机关公务用车制度改革，财税管理体制等改革也要取得新的进展。继续落实好国家赋予海南国际旅游岛的离岛免税、入境免签、探索发行竞猜型体育彩票等优惠政策，抓紧推动保税区建设，进一步强化以深海技术、遥感技术、南繁育种为基础的科技支撑，以南海佛学院为基础的文化支撑，以邮轮港和新机场为基础的硬件支撑，以旅游精品城市为载体的环境支撑，推动三亚早日建成国际门户枢纽机场和“一带一路”海上合作战略支点城市。抓紧研究我省获准开展服务贸易创新发展试点的政策优势，助推以旅游业为龙头的现代服务业快速向前发展。

（五）人民满意的政府必须是时刻倾听群众呼声的政府。我们要继续完善和发挥好12345、12301热线以及其他各种与市民游客联系沟通渠道的作用，随时了解群众的关切，第一时间回应群众的诉求，第一现场解决群众反映的问题。我们要密切关注网上舆情，高度重视媒体监督，将网上舆情监控纳入12345、12301平台，随时回应和解决网络及其他媒体反映的问题。我们要高度重视信访工作，强化信访事项督查督办，加强区一级的接访能力建设，就近解决群众的合理合法诉求。

（六）人民满意的政府必须是为民办实事的政府。我们要高度重视各项民生事业的发展，在扎实推进精准扶贫、促进居民就业增收、解决好菜价问题、加快教育和医疗卫生事业发展的同时，要不断优化社会保障体系，完善住房保障制度，支持社会救助体系建设和慈善事业发展，切实关心老年人、残疾人、贫困低保户等弱势群体的生活，进一步发展妇女儿童、青少年事业。要高度重视气象、地震工作和防灾减灾体系的建设，重视安全生产和食品药品安全工作，最大限度地减少各类自然灾害和安全事故给人民群众生命财产安全造成的损失。要高度重视文化事业的发展，通过建设各种文化设施、开展各种文化活动，积极整合、开发、利用和保护三亚的宗教、历史、红色、黎苗等本土文化，弘扬中华传统文化，吸收外来文化，让文化成为传承历史的纽带、对外沟通的桥梁。要高度重视驻市部队官兵和随军家属的生活，重视营区周边配套设施的建设和随军家属就业、子女入学等方面的安排，继续做好转业军人安置工作，坚持走军民融合发展的道路，切实抓好国防教育、国防动员、民兵预备役和双拥共建工作，以实际行动支持国防建设。

各位代表，如果说过去的五年是三亚实现跨越的五年，那么未来的五年必将是三亚实现腾飞的五年。让我们紧密团结在以习近平同志为核心的党中央周围，在省委、省政府和市委的坚强领导下，与全市人民一起，把握新常态，立足新起点，创造新业绩，为全面建成小康社会和国际化热带滨海旅游精品城市而努力奋斗，让三亚这颗镶嵌在祖国南海之滨的明珠焕发出更加夺目的光彩！
